# Prevention of Gastric Cancer: Eradication of *Helicobacter pylori* and Beyond

**DOI:** 10.3390/ijms18081699

**Published:** 2017-08-03

**Authors:** Tetsuya Tsukamoto, Mitsuru Nakagawa, Yuka Kiriyama, Takeshi Toyoda, Xueyuan Cao

**Affiliations:** 1 Department of Diagnostic Pathology, Fujita Health University School of Medicine, Toyoake 470-1192, Japan; nakaga1@fujita-hu.ac.jp (M.N.); ykiri@fujita-hu.ac.jp (Y.K.); 2 Division of Pathology, National Institute of Health Sciences, Tokyo 158-8501, Japan; t-toyoda@nihs.go.jp; 3 Department of Gastric and Colorectal Surgery, Jilin University, Changchun 130000, China; caoxueyuan19680414@yahoo.co.jp

**Keywords:** *Helicobacter pylori*, chronic atrophic gastritis, intestinal metaplasia, eradication, chemoprevention

## Abstract

Although its prevalence is declining, gastric cancer remains a significant public health issue. The bacterium *Helicobacter pylori* is known to colonize the human stomach and induce chronic atrophic gastritis, intestinal metaplasia, and gastric cancer. Results using a Mongolian gerbil model revealed that *H. pylori* infection increased the incidence of carcinogen-induced adenocarcinoma, whereas curative treatment of *H. pylori* significantly lowered cancer incidence. Furthermore, some epidemiological studies have shown that eradication of *H. pylori* reduces the development of metachronous cancer in humans. However, other reports have warned that human cases of atrophic metaplastic gastritis are already at risk for gastric cancer development, even after eradication of these bacteria. In this article, we discuss the effectiveness of *H. pylori* eradication and the morphological changes that occur in gastric dysplasia/cancer lesions. We further assess the control of gastric cancer using various chemopreventive agents.

## 1. Introduction

Although its prevalence is declining because of improved sanitation and antibiotic use, gastric cancer remains one of the leading causes of cancer-related deaths worldwide [1]. Thus, the prevention of gastric cancer is a substantial issue for cancer control programs. Various epidemiological, biological, and pathological characteristics of *Helicobacter pylori*-associated lesions have been evaluated in humans and animal models, especially in mice and Mongolian gerbils [2]. Recent health insurance program-supported efforts to eradicate *H. pylori* have been used for the prevention of gastric carcinogenesis, not only for patients with metachronous gastric cancer but also for those with chronic active gastric inflammation [3]. However, difficulties in demarcating cancerous lesions both endoscopically and histopathologically reveal that gastric cancer is still a major and challenging health issue [4]. In this article, we describe the challenges that exist in gastric cancer prevention strategies and compare human and animal lesions, with special attention to current pathological and biological findings.

## 2. Role of *H. pylori* Infection and Modifying Factors in Chronic Active Gastritis, Intestinal Metaplasia, and Gastric Carcinogenesis

### 2.1. Epidemiological Aspects

*H. pylori* was discovered in patients with chronic gastritis as Gram-negative, flagellated, microaerophilic bacilli, and was initially considered a species within the genus *Campylobacter* [5,6]. Strong clinical and epidemiological evidence has suggested that *H. pylori* is significantly correlated with active chronic gastritis, peptic ulcers, atrophic gastritis, intestinal metaplasia, and malignant lymphoma or cancer [7,8,9,10,11,12,13,14,15,16,17]. In a prospective study, Uemura et al. [18] confirmed that gastric cancer developed in only 2.9% of an *H. pylori*-infected symptomatic group compared to 0% in an uninfected group. The World Health Organization/International Agency for Research on Cancer evaluated *H. pylori* as a “definite biological carcinogen” based on epidemiological findings in 1994, requiring evidence of induction of gastric cancer in experimental animals [19].

### 2.2. Geographical Difference of H. pylori

*H. pylori* itself has several virulence factors. Among them, CagA has been reported to play an important role in gastric carcinogenesis. CagA is injected into gastric surface epithelial cells through the bacterial type IV secretion system, then is tyrosine-phosphorylated with Src and Abl [20] at variable EPIYA (Glu-Pro-Ile-Tyr-Ala) motif repeats region. These characteristic amino acids show structural diversity between East-Asian and Western countries [21]. *H. pylori*, found in the former, possess EPIYA-A, B, and D motifs, and the latter EPIYA-A, B, and C counterparts. Tyrosine phosphorylated EPIYA-C or D segments acquire the potential to interact with an oncoprotein, SHP2 phosphatase. East-Asian Cag A binds more strongly to SHP2 and induces morphological change, called the hummingbird phenotype, than does Western CagA [22]. This genetic variety may contribute to geographical difference for gastric carcinogenesis.

### 2.3. Animal Models

#### 2.3.1. Mouse Models

Several animal models have been used to mimic human gastric cancer caused by *H. pylori* infection, but most have yielded unsatisfactory results [23,24]. Human clinical samples infected with *H. pylori* were inoculated into nude and euthymic mice to determine the causative factor of chronic active gastritis [25,26,27]. In addition to *H. pylori*, another *Helicobacter* species, *H. felis*, is present in the cat stomach. This organism can be inoculated into germ-free mice to induce acute and chronic inflammation [28]. Lee et al. [29] established an *H. pylori* strain, the Sydney strain (SS1), which has been used frequently in mice. Recently, Draper et al. [30] compared the inter- and intra-genomic variability of two reference strains of *H. pylori*, PMSS1 (pre-mouse SS1) [31], a parental strain isolated from a human gastric ulcer patient, and SS1, a PMSS1 descendant being passed through mice for better mice colonization. The CagA copy number was noted as 1 and 4 in SS1 and PMSS1, correlating with the protein expression level. The most substantial alteration in the PMSS1 strain was an insertion in *cagY*, a *virB10* orthologue in the *cag* pathogenicity island (*cag*PAI) gene, which encodes a protein required for a type IV secretion system [32]. PMSS1 is now a valuable tool to study CagPAI, which requires the type IV secretion system [33].

Mice are resistant to a chemical carcinogen, *N*-methyl-*N*′-nitro-*N*-nitrosoguanidine (MNNG), which has been used to successfully induce gastric cancer in rats [34]. To study carcinogenesis in mice, investigators found that *N*-methyl-*N*-nitrosourea (MNU), another alkylating agent, could cause adenocarcinomas in the glandular stomachs of BALB/c [35] and C3H [36] mice. A gastric carcinogenesis model using this carcinogen was utilized in combination with *H. pylori* infection in later experiments to show that β-catenin activation may play an important role in distal carcinogenesis, especially in *H. pylori*-infected K19-C2mE transgenic mice compared to the non-treated K19-C2mE mice harboring predominantly proximal tumors. [37].

Genetic manipulation is used more successfully in mouse models than in other animal models [38]. To mimic *H. pylori*-induced inflammation, a transgenic mouse whose gastric epithelial cells simultaneously express both cyclooxygenase-2 (COX-2) and microsomal prostaglandin E synthase-1 under the control of the keratin 19 promoter, the K19-C2mE transgenic mouse, was established [39]. The combination treatment of K19-C2mE mice with MNU and *H. pylori* (Sydney strain, SS1) induced adenocarcinomas not only in the pyloric mucosa but also in the fundic glands, thus serving as a good model of proximal gastric cancer [37]. In addition to these inflammatory factors, the expression of Wnt1 was found to cause gastric lesions to become more dysplastic [40]. An interleukin-1β (IL-1β) polymorphism was reported to be involved in gastric carcinogenesis [41]. The overexpression of IL-1β, under the control of a parietal cell-specific H/K-ATPase promoter, caused transgenic mice to spontaneously develop chronic gastritis, intestinal metaplasia, and high-grade dysplasia/carcinoma with an accompanying *H. felis* infection compared to the control mice [42].

Ins-Gas mice harbor a chimeric insulin-gastrin (INS-GAS) transgene, in which the expression of the human gastrin gene is driven from the rat insulin I promoter [43]. Ins-Gas mice exhibited gastric metaplasia, dysplasia, carcinoma in situ, and gastric cancer with vascular invasion. *H. felis* infection accelerated cancer development with occasional submucosal invasion [44].

#### 2.3.2. Mongolian Gerbil Model

To better mimic severe human *H. pylori* infection and inflammation, a Mongolian gerbil (*Meriones unguiculatus*) model was successfully established. Infected animals develop chronic active gastritis, peptic ulcers, and intestinal metaplasia, resembling human lesions [45]. Twenty-five weeks after inoculation with *H. pylori*, the gastric glands become hyperplastic (heterotopic proliferative glands), characterized by severe chronic active gastritis with occasional penetrance through the muscularis mucosae. Fifty weeks after infection, intestinal metaplastic cells, including Alcian blue-stained goblet cells and/or absorptive cells that possess a striated brush border, appear among the gastric epithelial cells. After 75 weeks, the gastric cell phenotype gradually decreases, whereas the intestinal cell phenotype increases, accompanied by the formation of a more complete intestinal metaplasia, sometimes containing Paneth cells, by 100 weeks [46]. Heterotopic proliferative glands often appear resembling differentiated or mucinous adenocarcinomas because of their unusual structural abnormalities [47,48].

As in mouse models, chemical gastric carcinogenesis induced by MNU and MNNG can be modeled using Mongolian gerbils [49]. *H. pylori* infection accelerates both MNU- and MNNG-induced gastric carcinogenesis in a wide variety of cell types, including differentiated or signet-ring cell carcinomas [50,51,52].

### 2.4. Pathological Changes Caused by H. pylori Infection

In humans, chronic atrophic gastritis and intestinal metaplasia progress simultaneously. For the classification of intestinal metaplasia, we have proposed two categories [53]. The first is gastric-and-intestinal-mixed, which consists of atrophying gastric cells, including mucin core protein (MUC) 5AC-positive foveolar cells and/or MUC6-expressing pyloric cells, and intestinal cells, including MUC2-expressing/Alcian blue-stained goblet cells and CD10/villin-positive absorptive cells. These cells are putative markers of the progression of both chronic atrophic gastritis and intestinal metaplasia. The second category of intestinal metaplasia is the solely intestinal type, which is the extreme stage of intestinal metaplasia progression in this classification with disappearance of gastric compartments, accompanied by the full expression of intestinal markers, including caudal type homeobox 2 (CDX2), MUC2, and CD10. Thus, these subtypes of intestinal metaplasia reflect the gradual changes in gene expression during the progression from gastric to intestinal characteristics. This serial mucin change would cause spontaneous eradication of *H. pylori*, since the bacteria could colonize only in MUC5AC positive surface foveolar mucin but not in MUC2 positive intestinal mucins [2].

In the Mongolian gerbil model, gastric-and-intestinal-mixed type intestinal metaplasia was found to appear first, followed by the solely-intestinal type with the appearance of Paneth cells during the overall course of *H. pylori* infection [46]. Summarizing these human and animal data, intestinal metaplasia might be caused by the gradual intestinalization of gastric gland cells from the gastric-and-intestinal-mixed type to the solely-intestinal type.

Regarding stomach adenocarcinomas, gastric cancers at early stages mainly consist of gastric type cancer cells, and a phenotypic shift from gastric to intestinal phenotypic expression is observed with progression [53]. In the Mongolian gerbil gastric carcinogenesis model, 56 advanced glandular stomach cancers were analyzed for the gastrointestinal phenotypes. In *H. pylori*-infected gerbils, 56% (28 out of 50 cases) harbored the intestinal phenotype, but all the lesions (6/6) were classified as gastric type in non-infected gerbils. These findings suggested that adenocarcinomas also intestinalized with *H. pylori* infection and inflammation like intestinal metaplasia [54].

### 2.5. Host and Environmental Factors

Smoking has been shown to be associated with many kinds of human cancers [55]. For gastric cancers, a Japanese 10-year study has revealed that past and current smokers showed an increased risk of differentiated type gastric cancer in the distal region compared to non-smokers at a relative risk of 2.0 and 2.1, respectively [56]. A systematic review confirmed that relative risk for current smokers was estimated to be 1.56 (95% CI 1.36–1.80) for the Japanese population and concluded that tobacco smoking moderately increases the risk of gastric cancer, with the sex difference being 1.79 (1.51–2.12) and 1.22 (1.07–1.38) in men and women, respectively [57]. Tamer et al. [58] analyzed *glutathione S-transferases (GSTs)* genotypes in association with smoking and revealed that the *GSTM1* null genotype was associated with an increased gastric cancer risk for smokers (odds ratio (OR) = 2.15; 95% CI, 1.02–4.52), whereas no significant differences in the distributions of any of the other *GST* genes, *GSTT1* and *GSTP1*, existed in the Turkish population.

Males are at a higher risk of developing gastric cancer than females [59]. Androgen receptor in stromal cells was significantly higher in the advanced stage of gastric cancer in males, which might explain the gender difference [60].

### 2.6. Dietary Factors

#### 2.6.1. Salt

Among various food ingredients, salt and salted foods are probable risk factors for gastric cancer, based on evidence from a large number of case-control and ecological studies [61,62,63,64]. Tajima et al. [61] revealed that fondness for salted foods including pickled vegetable and dried and salted fishes, typical traditional Japanese foods, and showed a significantly positive association with stomach cancer at relative risk = 2.60. Several biologic markers in blood and urine were analyzed in ecological studies and revealed a significant and strong correlation between the amount of salt excreted in urine and stomach cancer mortality in both men and women in Japan [62], as well as worldwide [63].

Researchers have attempted to reveal how salted diet enhanced gastric carcinogenesis using an experimental model. In the pre-*Helicobacter* era, sodium chloride (NaCl) was found to enhance the carcinogenic effects of chemical carcinogens such as MNNG and 4-nitroquinoline 1-oxide (4-NQO) in the rat glandular stomach [65], possibly due to the reduction of the mucus viscosity and the impairment of the protective mucous barrier. Later, after the discovery of the bacteria in the human stomach, Nozaki et al. showed [66] how a high-salt diet enhanced the effects of *H. pylori* infection on gastric carcinogenesis. Although high salt intake alone had a minor influence on MNU induced gastric carcinogenesis, *H. pylori* infection and consequent inflammation acted synergistically with a high salt intake to promote the development of stomach cancers in the Mongolian gerbil model [67]. In *H. pylori-*infected gerbils, a high salt diet was associated with elevation of anti-*H. pylori* antibody titers, serum gastrin levels, and inflammatory cell infiltration in a dose-dependent fashion. The high salt diet upregulated the amount of surface mucous cell mucin, suitable for *H. pylori* colonization, but decreased the amount of gland mucous cell mucin, acting against *H. pylori* infection by inhibiting the bacterial cell wall component [68]. The incidences of glandular stomach cancers were 15% in the normal diet group and 33%, 36%, and 63% in the 2.5%, 5%, and 10% NaCl diet groups, showing a dose-dependent increase. The reduction of salt intake could thus be one of the most important strategies for the reduction of human gastric cancer.

#### 2.6.2. Green Tea

A comparative case-referent study revealed that the *OR* of stomach cancer decreased to 0.69 (95% confidence interval (CI) = 0.48–1.00) with a high intake of green tea (seven cups or more per day) [69]. A cross-sectional study was conducted on 636 subjects in Japan to examine the relationship among green tea consumption and *H. pylori*-induced chronic atrophic gastritis, and revealed that high green tea consumption (more than 10 cups per day) was negatively associated with the risk of chronic atrophic gastritis [70]. Many polyphenolic compounds have demonstrated anticarcinogenic activities, which included flavanone, flavonols, isoflavone, and catechins [71]. Epigallocatechin-3-gallate (EGCG), the major polyphenol in green tea, could affect carcinogenesis and the development of many cancers. Besides the anti-oxidative activity, EGCG inhibits the canonical Wnt/β-catenin signaling [72]. Ohno et al. [73] evaluated the protective effect of green tea catechins using Ins-Gas mice. Although catechin supplementation did not affect inflammation, dysplasia was significantly diminished histopathologically.

#### 2.6.3. Mastic Gum

Mastic gum is a resinous exudate obtained from *Pistacia lentiscus* which showed bactericidal activity against *H. pylori* in vitro [74]. An in vivo trial revealed no significant alleviation of *H. pylori* infection [75]. Another human trial illustrated the dose-dependent trend of mastic gum on *H. pylori* eradication, although this was not statistically significant [76].

In the mouse model infected with *H. pylori* SS1, the animals were administered with 2 g of mastic for seven days but failed to eradicate the infection [77]. In the other trial, administration of the total mastic extract without polymer at 0.75 mg/day to *H. pylori* SS1-infected mice for three months led to an approximate 30-fold reduction in the *H. pylori* colonization. However, no attenuation was observed in the *H. pylori*-associated inflammatory infiltration and the activity of chronic gastritis [78].

#### 2.6.4. Ginseng

Korean red ginseng extract, a herbal medicine, is widely used in Asian countries for various biological activities including its anti-inflammatory effect. Ginseng inhibits *H. pylori*-induced gastric inflammation in Mongolian gerbils by suppressing induction of inflammatory cytokines such as IL-1β, inducible nitric oxide synthase (iNOS), myeloperoxidase, and lipid peroxidase levels in *H. pylori*-infected gastric mucosa, although ginseng did not affect viable bacterial colonization in the stomach [79]. In vitro analysis revealed that Ginseng extract had strong anti-proliferative and pro-apoptotic effects on KATO3 human gastric cancer cells via the upregulation of Bax (B-cell lymphoma 2-associated X protein), IκBα (nuclear factor of kappa light polypeptide gene enhancer in B-cells inhibitor α) proteolysis, and the blocking of mTOR (mammalian target of rapamycin) and protein kinase B signaling [80]. In a case-control study, Yun et al. showed the preventive effect of ginseng intake against various human cancers including stomach cancer [81]. However, others did not illustrate clear results; further evaluation in Asian cohort studies may help clarify the role of ginseng in gastric carcinogenesis [82].

#### 2.6.5. Spices

*H. pylori* is known to play a causative role in gastric carcinogenesis, but wide variations in incidence have been noticed in Asian countries. *H. pylori* infection is more frequent in developing countries such as India, Pakistan, and Bangladesh than in other countries including Japan, China, and South Korea. Nonetheless, the frequency of gastric cancer is typically higher in the latter countries. This discrepancy is designated “the Asian enigma”, which may result from the genetic diversity of the infective *H. pylori* strains and differences in the genetic backgrounds of the various ethnic groups studied, as well as from their dietary habits [83]. To assess this problem, dietary spices were evaluated for the relief of *H. pylori*-induced inflammation. Capsaicin and piperine, but not curcumin, were found to have anti-inflammatory effects on *H. pylori*-induced gastritis in Mongolian gerbils, independent of direct antibacterial effects, and may thus function as chemopreventive agents for *H. pylori*-associated gastric carcinogenesis [84].

## 3. Effects of Eradication of *H. pylori* on Gastric Inflammation, Intestinal Metaplasia, and Carcinogenesis

### 3.1. Humans

Many researchers have attempted to clarify whether and how far the serial process of atrophic gastritis and intestinal metaplasia can be reversed after the eradication of *H. pylori.* As observed by endoscopic analysis, the enlarged or elongated pit patterns in *H. pylori*-positive specimens were improved to small, oval, pinhole-sized, or round pits after bacterial eradication, with decreased densities of fine, irregular vessels; such changes were not observed in specimens from subjects with severe gastric atrophy and intestinal metaplasia [85]. However, other reports have not always shown histological improvements in gastric atrophy and intestinal metaplasia after the eradication of *H. pylori* [86,87,88,89]. In contrast, some studies have reported that eradication effectively improves gastric lesions in the antrum or corpus [87,90,91,92].

**Figure 1:**
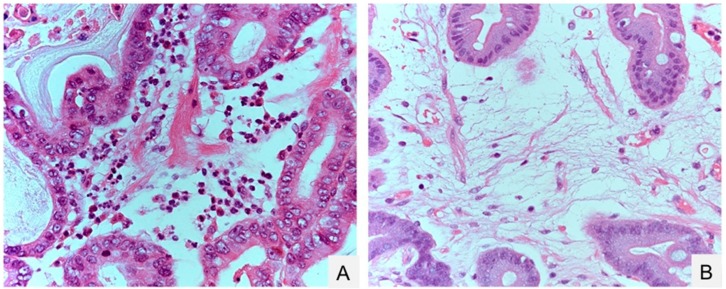
Gastric inflammation before and after eradication of *Helicobacter pylori*. (**A**) Neutrophil inflammation before *H. pylori* eradication; (**B**) edematous stroma after *H. pylori* eradication. Hematoxylin-Eosin (HE) staining. Original magnification, 400× (**A**,**B**).

After the eradication of *H. pylori*, the number of neutrophils drastically decreased, in contrast to the number of mononuclear cells, which gradually decreased (Figure 1). Eradication also alleviated the hyperplastic and hypertrophic enlargement of the surface foveolar epithelium in the gastric type, but not in the intestinal type, of metaplastic glands, as suggested by the endoscopic results mentioned above. It is currently unclear how bacterial eradication affects the amounts of the mucin core proteins, MUC5AC and MUC6, in these cells. In terms of morphological changes, the length of the proliferative zone and the number of Ki-67-positive cells were both significantly decreased in gastric-type glands [86,93] but not in intestinal metaplastic glands [86,94]. However, both gastric-and-intestinal-mixed and solely intestinal types of intestinal metaplasia always harbored larger numbers of mitotic cells, being positive for phosphorylated histone H3 protein at serine 28, than did gastric-type cells, regardless of the presence of *H. pylori* infection. Thus, the initial development of intestinal metaplasia could represent an irreversible change with atrophic gastritis [86].

The eradication of *H. pylori* has been approved for both the prevention of metachronous cancer and cases of chronic atrophic gastritis [3]. Long term follow up after treatment of *H. pylori* infection revealed the regression of preneoplastic gastric lesions, including intestinal metaplasia [95,96,97]. Both prospective [98,99] and retrospective [100] studies have documented that the successful eradication of *H. pylori* might reduce the occurrence of metachronous gastric cancer after the endoscopic resection of early lesions over a 3-year period. However, a 7.5-year randomized controlled trial in China revealed that the eradication of this organism significantly decreased the incidence of gastric adenocarcinoma in a subgroup of patients without atrophy, intestinal metaplasia, or dysplasia, whereas the overall incidence did not improve significantly between the eradication and placebo groups [101]. Another meta-analysis [102] supported the idea that eradication of *H. pylori* is only effective in a subgroup of patients without intestinal metaplasia or dysplasia. A prospective study monitored serum pepsinogen levels and the pepsinogen I/II ratio to determine the degree of chronic gastritis; these authors observed a significant reduction in cancer incidence in pepsinogen test-negative subjects with mild gastritis after *H. pylori* eradication over a mean period of 9.3 ± 0.7 years [103].

**Figure 2:**
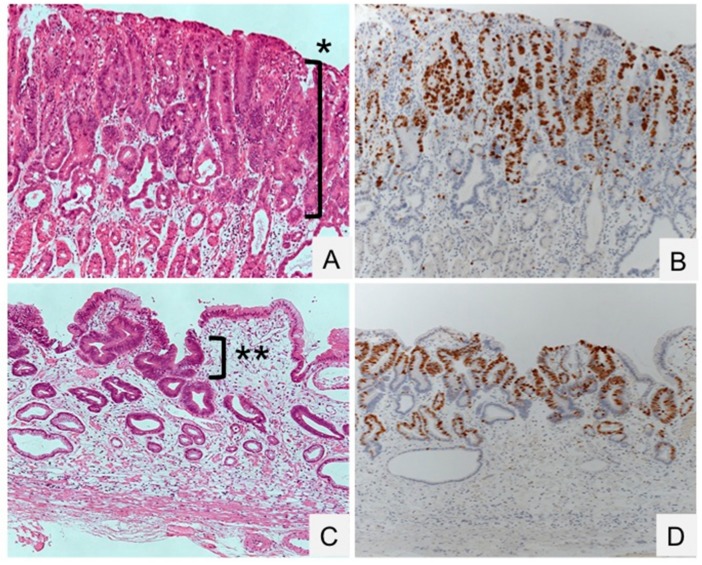
Gastric dysplasia (intramucosal adenocarcinoma) before and after eradication of *Helicobacter pylori*. (**A**,**B**) Dysplasia proliferating to the surface of the mucosa in an *H. pylori*-positive specimen (*). (**C**,**D**) Regression of dysplasia, localized beneath the normal surface epithelium in an *H. pylori*-negative specimen (**). HE staining (**A**,**B**) and Ki-67 immunostaining (**C**,**D**). Original magnification, 100×.

**Figure 3:**
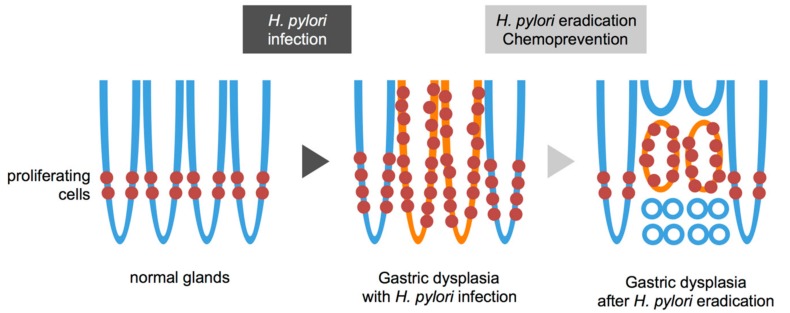
Schematic view of gastric dysplasia (intramucosal adenocarcinoma) before and after eradication of *Helicobacter pylori (H. pylori)*. Normal glands have proliferating cells in the lower narrow region (**left**). *H. pylori* infection widens proliferating zone in the normal glands (**middle**, **blue line**). Gastric dysplasia shows expanding proliferation with *H. pylori* infection and inflammation (**middle**, **orange line**). The tumor is shown around the proliferative zone (**right**, **orange line**) with subsequent regression at the top and bottom regions that are then occupied by adjacent normal epithelia (**right**, **blue line**) after eradication.

Endoscopic findings have revealed that the gastric tumor area has a gastritis-like appearance rather than typical malignant characteristics [4]. An histopathological analysis of gastric dysplasia (as in the Western category [104], which is intramucosal adenocarcinomas according to the Japanese criteria [105]) revealed significant and rapid alterations in tumor morphology and proliferative characteristics after the eradication of bacteria (Nakagawa et al., manuscript submitted) (Figure 2 and Figure 3). Additionally, gastric tumors appeared to be covered with normal [106] or low-grade atypical epithelium [107] after treatment with antibiotics. These morphological changes make the diagnosis of gastric dysplasia difficult using either endoscopic or histopathologic methods.

### 3.2. Animals

Several studies based on detailed histopathological assessments have reported a lack of carcinomas in animals subjected only to *H. pylori* infection [49,50,51,52,108]. With eradication therapy, the sizes of heterotopic proliferative glands were dramatically reduced, with only mucins remaining within them [46], indicating that *H. pylori* is a stronger promoter of gastric carcinogenesis than are carcinogens.

In the Mongolian gerbil model involving *H. pylori* infection and carcinogen treatment, *H. pylori* eradication provided direct evidence that gastric cancer can be prevented [108]. The incidence of adenocarcinoma was significantly lower after curative treatment of *H. pylori* infection than before treatment. Additional experiments using *H. pylori*-infected and carcinogen-treated Mongolian gerbils showed that earlier *H. pylori* eradication resulted in less carcinogenesis [109]. Animal models support the hypothesis that *H. pylori* eradication is useful for the prevention of gastric carcinogenesis, especially when performed during the early stages of cancer development.

## 4. Chemoprevention of Gastric Carcinogenesis

### 4.1. Oxygen Radical Scavengers

Natural products are believed to lower gastric cancer risk in humans [110]. Inflammation and subsequent oxidative stress play important roles in gastric carcinogenesis as mediators of DNA damage and carcinogen production [111]. The combination of bacterial eradication and the reduction of inflammation may be a more reasonable approach for the prevention of gastric cancer development, since the most important factor affecting gastric carcinogenesis is the severity of inflammation [112]. Using the Mongolian gerbil model, one of the most potent antioxidative compounds obtained from crude canola oil, 4-vinyl-2,6-dimethoxyphenol (canolol), was examined for its preventive effects against gastric inflammation and carcinogenesis in *H. pylori*-infected and carcinogen-treated animals. Canolol (0.1%) was mixed into food to suppress COX-2, iNOS, and 8-hydroxy-2′-deoxyguanosine, resulting in the marked reduction of the incidence of gastric adenocarcinoma, although the number of viable *H. pylori* was not changed [113]. Canolol also suppressed spontaneous gastric tumor development in K19-C2mE transgenic mice by reducing Cox-2, IL-1β, and IL-12β levels, possibly via the reactivation of tumor suppressor miR-7 microRNA [114]. Taking these results into account, the level of inflammation, rather than the existence of *H. pylori*, may be the most important factor in the process of carcinogenesis.

### 4.2. COX-2 Inhibitors

COX-2 and its downstream products play essential roles in the inflammatory microenvironment and tumorigenesis [115]. In mouse models, the overexpression of COX-2 has been shown to be associated with gastric and colorectal adenocarcinomas [37,39,40,116,117]. COX-2-selective inhibitors such as etodolac and celecoxib may have chemopreventive effects [118,119], not only suppressing inflammation but also causing tumor regression [120,121]. Considering the prevention of metachronous gastric cancer in patients that already have extensive metaplastic gastritis, COX-2 inhibitors could induce the regression of precancerous lesions and prevent gastric cancer occurrence after *H. pylori* eradication. In a nonrandomized trial, Yanaoka et al. [122] administered etodolac to serum pepsinogen test-positive and *H. pylori* antibody-negative patients, and found an effective reduction of metachronous cancer development. Another intervention trial with a COX-2 inhibitor, celecoxib, in combination with the eradication of *H. pylori* was conducted and showed the regression of gastric lesions, revealing the importance of the COX-2/prostaglandin E2 (PGE2) pathway [123].

## 5. Conclusions

Since the discovery of *H. pylori* in the human stomach, infection by these bacteria has been shown to be strongly associated with gastric lesions, including chronic atrophic gastritis, intestinal metaplasia, and gastric cancer. Epidemiological studies, in combination with results from animal models, confirm that eradication of *H. pylori* effectively prevents gastric carcinogenesis and mild gastritis without severe atrophy or intestinal metaplasia. However, bacterial eradication raises the issue of regression of gastric dysplasia (intramucosal adenocarcinoma), which might be underdiagnosed as a regenerating gland. Only by precise diagnoses, chemopreventive approaches, and *H. pylori* eradication can gastric cancer be conquered.
